# Microenvironmental Stiffness Enhances Glioma Cell Proliferation by Stimulating Epidermal Growth Factor Receptor Signaling

**DOI:** 10.1371/journal.pone.0101771

**Published:** 2014-07-07

**Authors:** Vaibhavi Umesh, Andrew D. Rape, Theresa A. Ulrich, Sanjay Kumar

**Affiliations:** Department of Bioengineering, University of California, Berkeley, California, United States of America; University of California, San Diego, United States of America

## Abstract

The aggressive and rapidly lethal brain tumor glioblastoma (GBM) is associated with profound tissue stiffening and genomic lesions in key members of the epidermal growth factor receptor (EGFR) pathway. Previous studies from our laboratory have shown that increasing microenvironmental stiffness in culture can strongly enhance glioma cell behaviors relevant to tumor progression, including proliferation, yet it has remained unclear whether stiffness and EGFR regulate proliferation through common or independent signaling mechanisms. Here we test the hypothesis that microenvironmental stiffness regulates cell cycle progression and proliferation in GBM tumor cells by altering EGFR-dependent signaling. We began by performing an unbiased reverse phase protein array screen, which revealed that stiffness modulates expression and phosphorylation of a broad range of signals relevant to proliferation, including members of the EGFR pathway. We subsequently found that culturing human GBM tumor cells on progressively stiffer culture substrates both dramatically increases proliferation and facilitates passage through the G1/S checkpoint of the cell cycle, consistent with an EGFR-dependent process. Western Blots showed that increasing microenvironmental stiffness enhances the expression and phosphorylation of EGFR and its downstream effector Akt. Pharmacological loss-of-function studies revealed that the stiffness-sensitivity of proliferation is strongly blunted by inhibition of EGFR, Akt, or PI3 kinase. Finally, we observed that stiffness strongly regulates EGFR clustering, with phosphorylated EGFR condensing into vinculin-positive focal adhesions on stiff substrates and dispersing as microenvironmental stiffness falls to physiological levels. Our findings collectively support a model in which tissue stiffening promotes GBM proliferation by spatially and biochemically amplifying EGFR signaling.

## Introduction

Glioblastoma (GBM) is the most commonly diagnosed primary astrocytoma in the United States and is also the most deadly primary brain tumor, with a median survival time of only 15 months [1]. Even with extensive resection, chemotherapy, and radiotherapy, recurrence occurs rapidly and almost universally but rarely involves extracranial metastasis. This suggests that signals encoded within the brain microenvironment may interact with cell-intrinsic factors to promote tumor progression, invasion, and recurrence, and that these cell-extrinsic signals may be investigated to achieve a more complete understanding of GBM and potentially uncover new therapeutic avenues [2], [3].

Of all of the microenvironmental parameters that may modulate GBM progression, mechanical signals remain among the most poorly understood. While it has long been understood that many tumors, including GBM, are mechanically stiffer than the surrounding stroma [4], [5], it has only recently become appreciated that these mechanical aberrations may actively instruct malignant progression rather than simply being a passive manifestation of tumor growth [6]–[8]. For example, we previously demonstrated that GBM cells show higher proliferation and migration rates when cultured on stiff two-dimensional substrates [9], [10]. Consistent with this idea, GBM tumors and culture models often display altered expression of molecules known to play key roles in sensing and/or responding to mechanical signals encoded in the tissue microenvironment (i.e., mechanosensing). This list includes integrins, which physically engage the extracellular matrix (ECM) and process mechanical inputs [11]–[13]; specific integrin subtypes have been implicated in GBM tumor initiation, with expression directly correlating with tumorigenicity [13]–[16]. Other members of the mechanosensing machinery have been similarly implicated in GBM growth and progression, including focal adhesion kinase (FAK) [17], [18], the Rho family GTPases [19], and nonmuscle myosin II [20], [21]. These findings are consistent with the broader recognition that aberrant mechanosensing may drive the progression of many solid tumors, including breast epithelial tumors [6].

At the same time, GBM is also closely associated with dysfunction in canonical mitogenic signaling, which in turn impacts proliferation, apoptosis resistance, and invasion. Most notably, amplifications and mutations in epidermal growth factor receptor (EGFR) represent one of the most common sets of genetic lesions in GBM, with EGFR amplifications present in up to perhaps 50% of GBM tumors [22], [23]. EGFR, also referred to as ErbB1 or HER1, is a member of the HER family of receptor tyrosine kinases [24]. Phosphorylation of downstream signaling molecules phosphoinositide 3-kinase (PI3K) and protein kinase B (PKB or Akt) by activated EGFR promotes cell proliferation [24], [25]. Importantly, amplification of the EGFR gene and expression of the EGFRvIII mutation are associated with significantly decreased overall survival [26], [27]. Due to the prominent role of EGFR in controlling the cell cycle and its correlation with poor prognosis, EGFR and EGFRvIII have recently emerged as promising therapeutic targets for the treatment of GBM [24], [28], as has PI3K [29]–[31].

Despite the established centrality of EGFR signaling to GBM progression and the recognition that GBM tumors are accompanied by profound changes in tissue stiffness, it is unknown what, if any, connections exist between these two classes of lesions. Specifically, does tissue stiffening modulate, potentiate, or otherwise interact with EGFR-based signaling to drive tumor cell proliferation? Evidence for such connections exists in breast tumors, with ErbB2 inhibition blunting ECM stiffness-induced promotion of malignancy in a mammary epithelial tumor culture model [6]. Conversely, integrin clustering induced by enhanced matrix cross-linking has been observed to amplify ErbB2-mediated Akt phosphorylation [32]. Together, these findings led us to hypothesize that microenvironmental stiffness cues can regulate GBM proliferation by modulating EGFR-based signaling [33], which we tested using a combination of defined-stiffness culture substrates, proteomic screens, proliferation and cell cycle analysis, and pharmacological loss-of-function studies. We find that microenvironmental stiffness amplifies proliferation, is associated with enhanced progression through the G1/S cell cycle checkpoint, and is accompanied by increased expression and/or activity of EGFR, Akt, and PI3K. We also find that EGFR and focal adhesion markers co-localize on stiff but not soft substrates, implying that stiffness may amplify these signals by physical clustering of EGFR. Our work offers direct evidence that mechanical signals are transduced through the EGFR pathway in GBM and support the emerging concept of synergy between mitogenic and mechanosensory signaling systems.

## Materials and Methods

### Cell Culture

U373-MG and U87-MG human glioma cells were cultured as previously described [9]. To clarify nomenclature, we obtained U373-MG cells from the University of California, Berkeley Tissue Culture Facility, which obtained these lines from the American Type Culture Collection (ATCC). Genomic analysis has revealed that ATCC U373-MG cells likely share origins with U251-MG glioma cells, [34] although meta-analyses indicate that these two lines have evolved into distinct entities with different karyotypes and drug sensitivities. [35] Briefly, cells were cultured in DMEM high glucose (1X) with L-glutamine without sodium pyruvate (Invitrogen) and supplemented with 10% Calf Serum Advantage (JR Scientific, Inc.), 1% penicillin/streptomycin, 1% MEM nonessential amino acids (Invitrogen), 1% sodium pyruvate (Invitrogen). Cells were maintained in a humidified incubator at 37°C and 5% CO_2_.

### Synthesis of ECM substrates

Polyacrylamide substrates ranging from 0.08 kPa–119 kPa were fabricated as described previously [9]. Briefly, acrylamide solution (Bio-Rad) ranging from 3%–15% was mixed with N-N'- methylene-bis-acrylamide solutions (Bio-Rad) ranging from 0.05%–1.2% and then polymerized between a glutaraldehyde-activated glass surface and hydrophobic coverslip using 10% ammonium persulfate (Bio-Rad) and 1/2000 TEMED (Sigma-Aldrich). Polymerized substrates were then activated for protein conjugation with the water-soluble, heterobifunctional crosslinker Sulfo-SANPAH at 0.5 mg/mL (Pierce Chemical Co.) under UV exposure followed by functionalization with human plasma fibronectin (Millipore Corp.) at a nominal surface density of 2.6 µg/cm^2^.

### Flow cytometric studies

Glioma cells were plated on fibronectin-coated polyacrylamide substrates at a density of 10000 cells/cm^2^ (on 119 kPa substrates) and 20000 cells/cm^2^ (on 19 kPa, 0.8 kPa, and 0.08 kPa substrates). After ∼24 hours of incubation, cell proliferation was then measured according to the FITC-bromodeoxyuridine (BrdU) flow kit protocol (BD Biosciences) with a 90-minute exposure to 5-BrdU. Samples were then analyzed on a flow cytometer FC500 (Beckman-Coulter). An aggregate distribution of cells were gated on an FL4 (7-AAD) channel vs. FL1 (FitC-BrdU) channel plot and BrdU intensity was quantified relative to a non-BrdU treated (negative) control for each condition. The percent of BrdU positive cells was reported as the percent of proliferating cells in a given sample.

### Cell cycle analysis

Glioma cells were cultured on the surface of fibronectin-coated polyacrylamide gels for 48 hours prior to trypsinization, fixation, and staining with propidium iodide to quantify DNA content. Cells were then analyzed on a flow cytometer FC500 (Beckman-Coulter). An aggregate distribution of cells was visualized using a histogram of PI intensities and gated to exclude unviable cells and doublets. The gated population was visualized as a histogram and fit to the Watson model to quantify the percent of cells in the G0/G1, S and G2 phases of the cell cycle.

### Western blot

U373-MG and U87-MG cells were cultured on fibronectin-coated polyacrylamide substrates of defined stiffness for 48 hours. Cells on each substrate were washed twice in PBS, collected, and lysed using 50 µL RIPA lysis buffer with protease inhibitor (1∶100, Sigma-Aldrich) and phosphatase inhibitor (1∶100, Calbiochem) for 5 minutes. Proteins from cell lysates were separated using standard sodium dodecyl sulfate (SDS)-polyacrylamide gel electrophoresis (PAGE) and electrophoretically transferred to polyvinylidene fluoride (PVDF) membranes. Immunoblots were performed according to manufacturer specifications (Invitrogen Western Blot kit) as described in a previously established protocol (34). Following blocking, sections of the membrane containing the protein of interest were blotted with the appropriate primary antibody (overnight, 4°C) followed by a horseradish peroxidase-conjugated secondary antibody (1 hour at room temperature) prior to detection by chemiluminescence (West Dura). After development and scanning, band intensities were quantified by ImageJ (NIH). Primary antibodies included: EGFR (1∶500, Santa Cruz Biotechnology Inc.), P-EGFR (1∶1000, Cell Signaling) Akt (1∶40,000, Cell Signaling), P-Akt (1∶20,000, Cell Signaling) PI3K (1∶40,000, Cell Signaling), GAPDH (1∶5,000,000, Sigma-Aldrich).

### Immunostaining

U373-MG human GBM cells were seeded on polyacrylamide substrates of varying stiffnesses and allowed to equilibrate overnight before fixation. Cells were fixed with 4% paraformaldehyde in PBS, permeabilized using 0.1% Triton-X100 in PBS, and blocked using 5% goat serum in PBS prior to staining with the appropriate antibodies: P-EGFR (1∶250, Cell Signaling), Vinculin (1∶250, Sigma), DAPI (1∶200, Invitrogen). All fluorescence imaging were performed on a Prairie SFC confocal microscope

### Pharmacologic inhibitor studies

U373-MG cells were cultured on fibronectin-coated polyacrylamide substrates of varying stiffness for at least 24 hours prior to treatment with a single pharmacologic inhibitor. Cell proliferation was measured 24 hours post treatment with the drug of interest according to the FitC-BrdU flow kit protocol as described above. Pharmacologic inhibitors included: Tyrphostin AG1478 (20 uM, Calbiochem), Triciribine (20 uM, Enzo Diagnostics), Wortmannin (20 uM, Sigma-Aldrich).

### Reverse phase proteomic analysis (RPPA)

U373-MG and U87-MG cells were seeded on fibronectin-coated polyacrylamide substrates of varying stiffnesses. Total cellular protein was isolated using lysis buffer provided by the MD Anderson RPPA Core Facility and then sent to that facility for completion of RPPA following standard protocols (http://www.mdanderson.org/education-and-research/resources-for-professionals/scientific-resources/core-facilities-and-services/functional-proteomics-rppa-core/education-and-references/index.html).

## Results

### Microenvironmental stiffness influences proliferative signaling in glioma cells

To broadly explore whether stiffness-induced signals may influence the activity of proteins relevant to mitogenic signaling and proliferation, we harvested lysates from human GBM cells cultured on ECM protein-coated substrates of defined stiffness (from 0.08 kPa to 119 kPa) and used reverse phase protein array (RPPA) analysis to comparatively measure levels of a variety of proteins and phosphoproteins. In this technology, cell lysates are immobilized as spots onto a solid support, and each spot is probed with a distinct primary antibody directed against a known molecular target. Each spot is then incubated with a single biotin-tagged secondary antibody, which is then fluorescently labeled for quantification of target abundance. This approach therefore enables the parallel quantification of a large number of protein and phosphoprotein targets from the same lysate. We cultured U373-MG and U87-MG human glioma cells on substrates ranging from brain-like (0.08 kPa) to supraphysiological stiffness values (119 kPa) for 2 days in growth medium, harvested lysates, subjected the lysates to RPPA measurement, and analyzed the resulting data to identify proteins and phosphoproteins whose levels correlated significantly with stiffness (Spearman correlation coefficient R>0.5). Out of the 200 antibody targets that were probed by RPPA, the abundance of 48 correlated positively with stiffness for both U373-MG and U87-MG cells (Figure S1). Interestingly, more than one-quarter (∼27%) of these positive targets fell within pathways canonically associated with proliferation, including MAPK, RAF1, and Src (Figure S1; highlighted). Notably, EGFR levels were found to be significantly correlated with stiffness in U373-MG cells (r = 0.8205), as were the levels of two EGFR phosphoisoforms associated with EGFR auto-phosphorylation (Figure S1; r = 0.5830 and r = 0.5614 for pY106 and pY117, respectively). Finally, phosphorylation levels of the downstream EGFR signaling targets Akt and PI3K were strongly correlated with stiffness in both U373-MG (r = 0.8421 and 0.8205) and U87-MG cells (r = 0.5398 and 0.8205).

### Microenvironmental stiffness regulates human glioma cell proliferation

**Figure 1:**
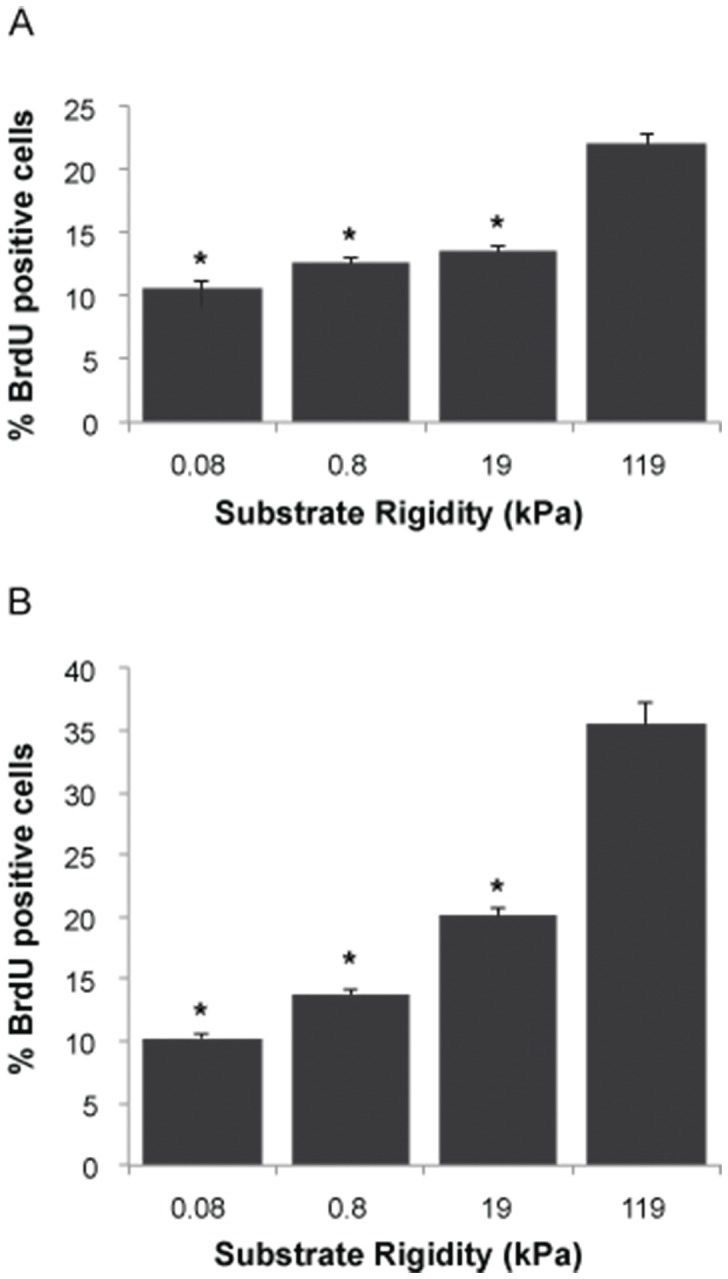
Microenvironmental stiffness regulates glioma cell proliferation. Effect of ECM rigidity on proliferation of U373-MG (A) and U87-MG (B) cells. Results represent quantification of n>10,000 cells for at least three substrates per condition by flow cytometry, where the percentage of dividing cells was determined as the average percentage of cells staining positive for BrdU incorporation. *, P<0.05 with respect to 119 kPa.

Given the broad correlations between substrate stiffness and the abundance of proteins and phosphoproteins associated with proliferation-related signaling, we next decided to directly quantify the extent to which substrate stiffness regulates proliferation. In a previous study [9] we showed that human GBM cells cultured on stiff substrates proliferated much more avidly than cells on highly compliant substrates of elasticity comparable to normal brain tissue. Because of the limited throughput and precision of the immunofluorescence-based bromodeoxyuridine (BrdU) incorporation method used in this earlier study, we first confirmed this result using a flow cytometry-based BrdU incorporation assay, which enables rapid analysis of tens of thousands of cells (Figure 1). We cultured cells on fibronectin-conjugated polyacrylamide hydrogels, transiently pulsed them with BrdU, harvested them from the substrate, and then measured the fraction of BrdU-positive cells by flow cytometry. Gradually increasing ECM stiffness from 0.08 kPa to 119 kPa dramatically enhanced proliferation in both U373-MG (Figure 1A) and U87-MG (Figure 1B) cells, with the stiffest ECM producing 2-3-fold more BrdU-positive cells than the softest ECM.

### Stiff microenvironments enhance progression through the G1/S checkpoint of the cell cycle

**Figure 2:**
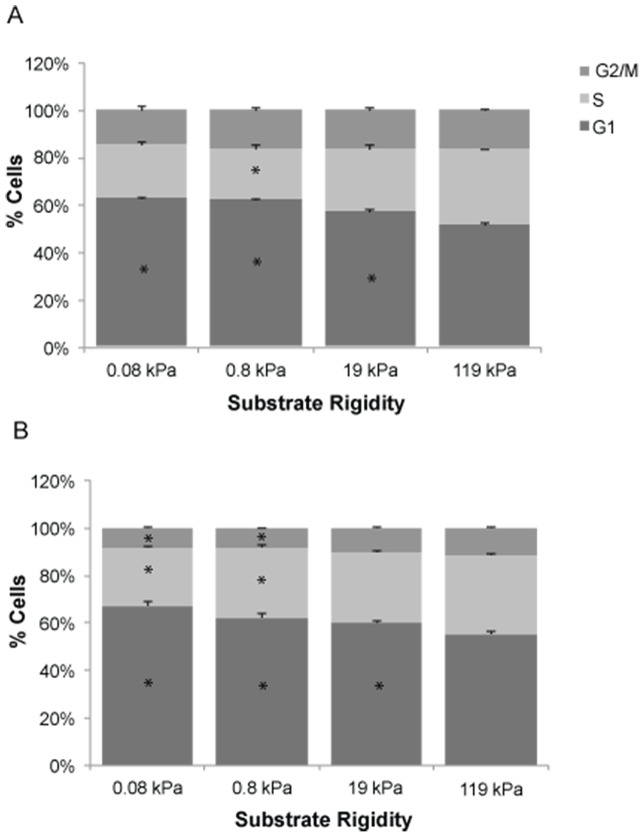
ECM rigidity regulates glioma cell cycle distribution. Effect of ECM rigidity on cell cycle distribution of U373-MG (A) and U87-MG (B) cells. Results represent quantification of n>10,000 cells for at least three substrates per condition by flow cytometry, where the percentage of cells in each phase of the cell cycle was determined as the average percentage of cells staining positive for propidium iodide incorporation. *, P<0.05 with respect to 119 kPa.

To gain additional mechanistic insight into the dramatic increase in cell proliferation induced by microenvironmental stiffness, we asked whether this effect might be accompanied by changes in cell cycle distribution. We therefore performed additional flow cytometric studies in which we cultured GBM tumor cells on a range of defined-stiffness substrates, treated the cells with propidium iodide to mark DNA content, and performed flow cytometry to measure distribution across the G1, S, and G2/M phases of the cell cycle (Figure 2). For both U373-MG (Figure 2A) and U-87 MG (Figure 2B) cells, the majority of cells were in G1 phase across all stiffness values (Figure 2; dark gray). Interestingly, however, we noted that increasing ECM stiffness increased the percentage of cells in S phase (Figure 2; light gray), with concomitant reductions in the number of cells in G1. For U87-MG cells on the two softest ECMs considered, there was also a corresponding depletion of cells in G2/M phase. Taken together with our proliferation data (Figure 1), these results are consistent with a mechanism in which increasing microenvironmental stiffness accelerates proliferation by facilitating passage through the G1/S checkpoint.

### Increasing microenvironmental stiffness promotes expression and phosphorylation of EGFR-induced signals

**Figure 3:**
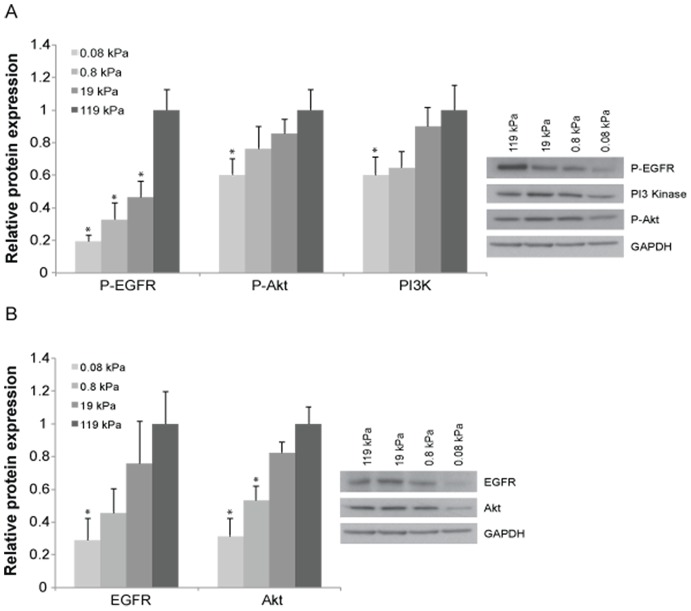
Microenvironmental stiffness regulates expression and phosphorylation of EGFR pathway components. The expression of activated EGFR, activated Akt and PI3K in U373-MG cells rises with increasing substrate stiffness (A). Similarly, the expression levels of EGFR and Akt in U373-MG cells rise with increasing substrate stiffness (B). Results represent quantification of at least three biological replicates on three separate Western blots, where the relative protein expression levels have been first normalized to the expression of GAPDH and then normalized to the expression level on the stiffest substrate of 119 kPa. Representative blots for each protein are on the right. *, P<0.05 with respect to 119 kPa.

Our flow cytometry results motivated us to consider potential molecular mechanisms through which increasing ECM stiffness might speed passage through the G1/S checkpoint. EGFR activation is known to promote proliferation in part by accelerating G1/S passage [36]–[39] and is among the most commonly aberrant genes in GBM. Given this and our RPPA finding that substrate stiffness is correlated with expression and phosphorylation of EGFR signaling proteins in both U373-MG cells (EGFR, pEGFR, Akt, pAkt, pPI3K) and U87-MG cells (Akt, pAkt, pPI3K), it occurred to us that ECM stiffness might act through EGFR signaling to promote cell cycle progression and proliferation [22]. However, an important caveat of RPPA is its comparatively limited dynamic range and sensitivity [40], which suits RPPA well for detecting broad correlations among experimental parameters but much less so for quantification of protein levels. To more precisely and quantitatively measure these potential stiffness-dependent proteomic changes, we used Western blots to determine if microenvironmental stiffness could alter expression or phosphorylation of EGFR or its downstream effectors Akt and PI3K (Figure 3A). Remarkably, increasing matrix stiffness from 0.08 kPa to 119 kPa produced a five-fold increase in phosphorylated EGFR (pEGFR) and nearly two-fold increases in phosphorylated Akt (pAkt) and total PI3K. Increasing matrix stiffness over this same range also strongly increased overall levels of EGFR and Akt (Figure 3B), suggesting that the enhancement of phosphorylation may result in part from greater overall levels of each protein. Importantly, these studies were conducted in the absence of exogenous EGF beyond levels already present in serum or secreted by cells. Thus, increasing microenvironmental stiffness broadly activates EGFR signaling in GBM tumor cells.

### EGFR pathway inhibition renders proliferation significantly less sensitive to substrate stiffness

**Figure 4:**
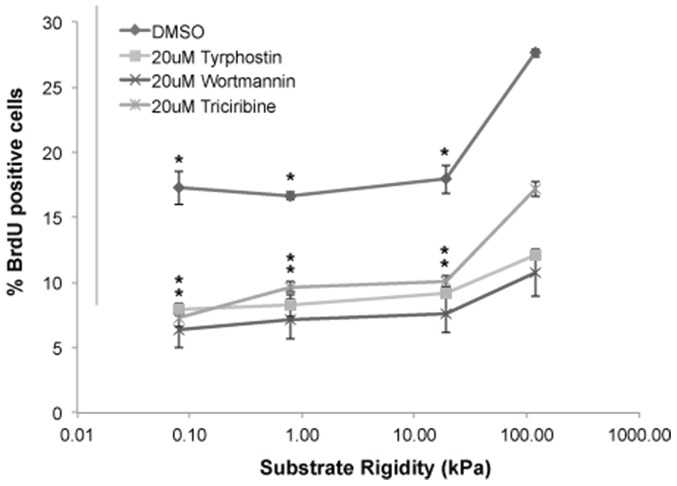
Stifffness-dependent glioma cell proliferation is dampened upon treatment with 20 uM EGFR inhibitor - Tyrphostin, 20 uM Akt inhibitor - Triciribine, and 20 uM PI3 Kinase inhibitor - Wortmannin for 24 hours as compared with the DMSO negative control. Results represent quantification of n>10,000 cells for at least three substrates per condition by flow cytometry, where the percentage of dividing cells was determined as the average percentage of cells staining positive for BrdU incorporation *, P<0.05 with respect to 119 kPa for DMSO control, 20 uM Tyrphostin and 20 uM Triciribine.

The above results indicate that increasing matrix stiffness enhances cell proliferation (Figure 1), facilitates passage through the G1/S checkpoint (Figure 2), and potentiates EGFR pathway activation (Figure 3A). To determine whether EGFR pathway activation is necessary for stiffness-induced proliferation, we performed studies in which we cultured cells on defined-stiffness substrates, treated them with pharmacologic inhibitors of EGFR kinase (Tyrphostin), Akt kinase (Triciribine), PI3K (Wortmannin), or, due to the known effects of DMSO on proliferation [41], [42], a DMSO-only control. The specificity and efficacy of these drugs have been extensively characterized in previous studies [43]–[45]. We then repeated BrdU flow cytometric analysis to determine EGFR pathway-dependent effects on cell proliferation. As expected, DMSO-treated U373-MG controls strongly exhibited stiffness-dependent proliferation as observed earlier (Fig. 1). However, treatment with any of the three inhibitors both reduced overall levels of proliferation and desensitized proliferation to stiffness, with the strongest effect observed for EGFR and PI3K inhibition (Figure 4). Thus, GBM tumor cell proliferation is substantially less sensitive to ECM stiffness when EGFR signaling is reduced, implying that matrix stiffness acts in part through EGFR-mediated signaling pathways to promote proliferation.

### Changes in microenvironmental stiffness alter EGFR organization and co-localization with focal adhesions

**Figure 5:**
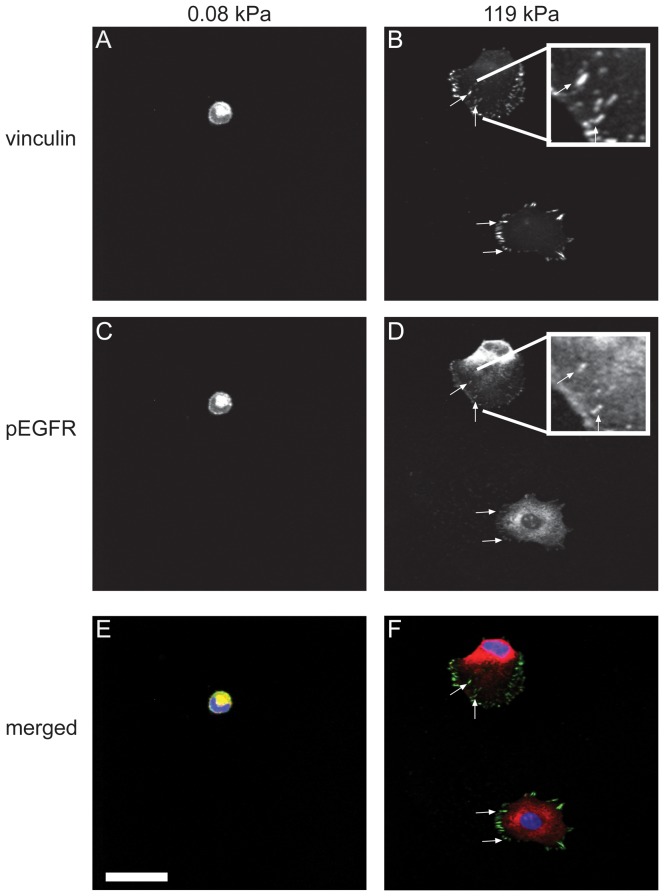
Colocalization of focal adhesion of phospho-EGFR. U373-MG cells were cultured on soft (A, C, E) or stiff (B, D, F) polyacrylamide hydrogels and immunofluorescently stained for vinculin (A, B) and phospo-EGFR (C, D). There are no punctate vinculin-positive focal adhesions on soft substrates (A), while there are large focal adhesions on stiff substrates (C). On stiff substrates, there are distinct, punctate pEGFR structures (D; arrows) that colocalize with vinculin positive adhesions (F; arrows). The colocalization is more clearly evident in the high-magnification insets (B, D). Scale bar is 50 microns.

Modulation of tissue stiffness is widely understood to control cell physiology through a number of proximal signals, perhaps the most well-studied of which is assembly of integrin-based adhesion complexes. These adhesions can influence growth factor signaling in a number of important ways, including locally concentrating growth factor receptors and recruiting key mitogenic signaling intermediates such as focal adhesion kinase (FAK) and PI3K. This is important in that EGFR activation is strongly amplified by spatial clustering of the receptor and its downstream effectors [46]. To determine whether matrix stiffness might influence the assembly of EGFR, we cultured U373-MG cells on defined-stiffness matrices and used immunofluorescence to examine colocalization of EGFR and focal adhesion proteins (Figure 5). As expected from our and others' previous studies [9], [47], soft ECMs gave rise to immature, punctate vinculin-positive focal complexes, with stiffer ECMs yielding larger and more elongated focal adhesions. Strikingly, these changes in substrate stiffness also concomitantly enhanced pEGFR clustering, with pEGFR strongly co-localizing with vinculin-positive focal adhesions and forming large structures on stiff matrices. Thus, increasing microenvironmental stiffness promotes the clustering and colocalization of both integrin-based focal adhesion complexes and pEGFR.

## Discussion

Our study shows that microenvironmental stiffness increases the expression and/or phosphorylation of EGFR and its downstream effectors, and that stiffness-dependent signals stimulate proliferation by acting in part through EGFR-based mitogenic signaling. While the precise mechanism of this interaction remains to be fully elucidated, the strong, stiffness-dependent colocalization of pEGFR with focal adhesion components is consistent with a model in which tissue stiffening promotes GBM proliferation by spatially and biochemically amplifying EGFR signaling. If this is the case, then mechanotransductive and EGFR-based signals may act synergistically to regulate cell proliferation in GBM.

Previous research has suggested the possibility of cooperativity between mechanical inputs and growth factor signaling. Many growth factor receptors including EGFR can interact both directly and indirectly with a variety of integrin subtypes and colocalize within integrin-based adhesions [48]–[51]. Moreover, studies using both in vitro and mouse models of various tumors have suggested that integrin clustering and matrix stiffness may be at least partially responsible for enhanced PI3K signaling. For example, inhibition of PI3K signaling was found to neutralize the tumor-promoting effects of matrix stiffness in a mouse model of breast cancer [32]. Furthermore, reducing substrate stiffness normalized invasive, disorganized colonies formed by EGFR-transformed mammary epithelial cells cultured in reconstituted basement membrane matrices [6]. Moreover, changes in matrix stiffness have previously been shown to alter cell cycle progression in mammary epithelial cells, smooth muscle cells, fibroblasts, and other non-neuroglial cell types [33], [52]. While this body of work implies fundamental connections between growth factor receptors, their canonical downstream targets, and mechanotransductive signaling systems in regulating tumor propagation and invasion, relatively little is known regarding the underlying phenotypic mechanisms or if these findings extend to other tumors. Our results begin to fill this gap by supporting the notion that EGFR- and mechanotransductive signaling act in tandem to promote proliferation in GBM cells, although further investigations are necessary to determine if this is a general phenomenon of mechanosensing or is specific to GBM and perhaps other tumor types. A key limitation of our studies is the use of highly reductionist culture models, which was necessary to cleanly isolate stiffness as an experimental variable. However, future studies in which EGFR and mechanotransductive signals are simultaneously manipulated in vivo (e.g. in orthotopic xenograft paradigms) should help clarify the physiological role of this phenomenon and the relative influence of other inputs that may modulate PI3K/Akt signaling in vivo. These studies would also serve as an important check against our pharmacological inhibition studies, where legitimate concerns may exist about target specificity.

We show that focal adhesions and EGFR co-localize on stiff but not soft substrates, suggesting that enhanced EGFR clustering on stiff substrates may be driven in part by interactions between focal adhesion proteins and EGFR. Importantly, forced clustering of EGFR mutants enhanced tumorgenicity and decreased survival time in a mouse xenograft model of GBM [53]. Much previous work supports the existence of interactions between focal adhesion proteins and EGFR, with many of these efforts focusing specifically on the interaction between focal adhesion kinase (FAK) and EGFR. FAK is a ubiquitously expressed tyrosine kinase that contains an N-terminal FERM domain and a C-terminal focal-adhesion targeting domain (FAT) [54]. The FERM domain has been shown to bind to certain growth factor receptors, including EGFR, while the FAT domain causes FAK to localize to focal adhesions. Focal adhesion-localized EGFR then signals directly through the Band-4.1 domain on FAK, thereby providing a direct link between known mechanosensory machinery and the EGFR pathway [49]. The importance of this connection is highlighted by experiments in mouse models of breast cancer, where FAK is required for ErbB2/3 mediated oncogenic transformation and lung metastases of MDA-231-M2 cell injected into the mammary fat pad [55]. This FAK-based connection may have clinical significance given that FAK inhibition was recently shown to sensitize GBM cells to PD153035-induced EGFR inhibition [56]. Thus, in the future it should be valuable to more precisely dissect the role of FAK in coupling mechanotransductive and EGFR-dependent control of GBM proliferation.

One somewhat unexpected finding from our study is that increases in microenvironmental stiffness increase total levels of EGFR and its downstream effectors, in addition to levels of the corresponding phosphoproteins. This implies that substrate stiffness may influence the transcription, translation, and/or degradation of these proteins. While surprising, similar effects have been observed in breast tumor cells grown in three-dimensional reconstituted basement membrane, where EGFR overexpression has been found to trigger compensatory α1 integrin upregulation [48]. Similarly, total and phospho-EGFR levels are reduced when cytoskeletal tension is relaxed in mammary epithelial cells [6]. Numerous other studies have established a link between mechanotransductive signaling and transcriptional regulation [57]–[59], in which activation of mechanotransductive signals at the plasma membrane may influence transcription through traditional signal transduction events or more hypothetically through direct mechanical deformation of the nucleus [60], [61].

In conclusion, we have investigated interactions between microenvironemental stiffness and EGFR-dependent signaling in controlling cell cycle and proliferation. Our data are broadly consistent with a model in which stiffness enhances EGFR-dependent signaling to regulate proliferation. As GBM tumors are known to be stiffer than normal brain tissue, these stiffness changes may modulate cell proliferation in vivo. An important limitation in making this connection in a more literal way is the relative absence of quantitative measurements of tumor stiffness, which remains technically challenging. As these values become available, it will be informative to revisit these studies with materials designed to tightly bracket that range. Finally, while it may be premature to speculate on the clinical implications of this finding, our results raise the interesting possibility that modulation of microenvironmental mechanics and/or mechanotransductive signaling systems may be used to potentiate the effects of EGFR and PI3K inhibitors. While these small-molecule inhibitors have shown great promise in preclinical studies and early clinical trials, much room certainly remains for improvement [62]–[64]. Analogously, integrins are under evaluation as therapeutic targets in GBM, with an RGD peptide inhibitor showing modest increases in progression-free survival in phase II clinical trials and failed to do so in phase III trials [65], [66]. Co-administration of EGFR pathway inhibitors and agents that modulate the mechanotransduction machinery may thus enhance the activity of both agents. There is ample precedent for such co-administration strategies; for example, in vivo mouse studies have suggested that using Y15, a FAK autophosphorylation inhibitor, synergistically with temozolomide is a more effective at preventing tumor growth than either drug alone [17]. It will be important to carefully and systematically evaluate these concepts in both primary human GBM xenografts and other preclinical models.

## Supporting Information

Figure S1 **Microenvironmental stiffness-dependent regulation of proteins in U373-MG and U87-MG cells.** U373-MG and U87-MG human glioma cells were cultured on one of four defined-stiffness substrates and then subjected to reverse phase protein array (RPPA) analysis. Correlations between substrate stiffness and protein expression were quantified by Spearman correlation analysis for each cell type. A significant correlation is defined as a correlation coefficient (R) of absolute value greater than 0.5. The table includes only proteins whose levels correlate significantly with stiffness in both U373-MG and U87-87 cells, U373-MG only, or U87-MG only. For proteins that correlate significantly with both cell lines, R values are the reported as the average of the absolute values of the R values for the individual cell lines. All other R values are reported as the absolute vale of the R score. Proteins known to be related to proliferation are highlighted in yellow.
(DOCX)
