# Supplementary material for: Microenvironmental Stiffness Enhances Glioma Cell Proliferation by Stimulating Epidermal Growth Factor Receptor Signaling
Source: PLoS One. 2014 Jul 7;9(7):e101771. doi: 10.1371/journal.pone.0101771 (PMC4084995; doi:10.1371/journal.pone.0101771)
Supplement: Figure S1 — Microenvironmental stiffness-dependent regulation of proteins in U373-MG and U87-MG cells. U373-MG and U87-MG human glioma cells were cultured on one of four defined-stiffness substrates and then subjected to reverse phase protein array (RPPA) analysis. Correlations between substrate stiffness and protein expression were quantified by Spearman correlation analysis for each cell type. A significant correlation is defined as a correlation coefficient (R) of absolute value greater than 0.5. The table includes only proteins whose levels correlate significantly with stiffness in both U373-MG and U87-87 cells, U373-MG only, or U87-MG only. For proteins that correlate significantly with both cell lines, R values are the reported as the average of the absolute values of the R values for the individual cell lines. All other R values are reported as the absolute vale of the R score. Proteins known to be related to proliferation are highlighted in yellow. (DOCX) [file pone.0101771.s001.docx]

**Supplemental Figure S1**


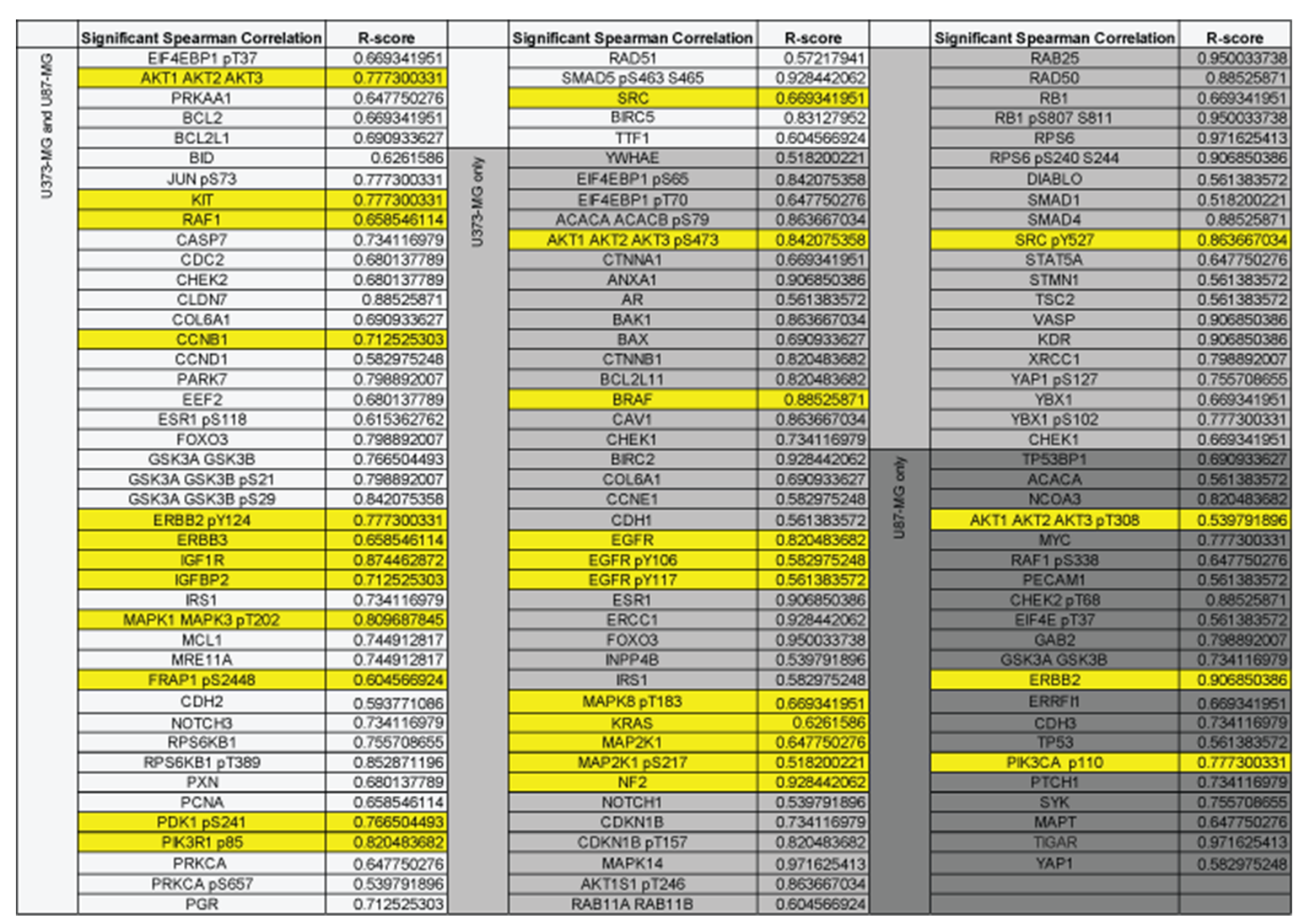


**Figure S1.** Microenvironmental stiffness-dependent regulation of proteins in U373-MG and U87-MG cells. U373-MG and U87-MG human glioma cells were cultured on one of four defined-stiffness substrates and then subjected to reverse phase protein array (RPPA) analysis. Correlations between substrate stiffness and protein expression were quantified by Spearman correlation analysis for each cell type. A significant correlation is defined as a correlation coefficient (R) of absolute value greater than 0.5. The table includes only proteins whose levels correlate significantly with stiffness in both U373-MG and U87-87 cells, U373-MG only, or U87-MG only. For proteins that correlate significantly with both cell lines, R values are the reported as the average of the absolute values of the R values for the individual cell lines. All other R values are reported as the absolute vale of the R score. Proteins known to be related to proliferation are highlighted in yellow.
